# Brain Connectivity of the Cingulate Cortex in Alcohol Use Disorder: Exploring Its Association With Mindfulness

**DOI:** 10.1111/adb.70036

**Published:** 2025-10-10

**Authors:** Niklaus Denier, Kevin Zahnd, Maria Stein, Franz Moggi, Zeno Kupper, Andrea Federspiel, Roland Wiest, Matthias Grieder, Leila M. Soravia, Tobias Bracht

**Affiliations:** ^1^ Translational Research Center, University Hospital of Psychiatry and Psychotherapy University of Bern Bern Switzerland; ^2^ Translational Imaging Center (TIC) Swiss Institute for Translational and Entrepreneurial Medicine Bern Switzerland; ^3^ Department of Clinical Psychology and Psychotherapy, Institute of Psychology University of Bern Bern Switzerland; ^4^ Institute of Diagnostic and Interventional Neuroradiology University of Bern Bern Switzerland; ^5^ University Hospital of Forensic Psychiatry and Psychology University of Bern Bern Switzerland; ^6^ Clinic Suedhang Bern Switzerland

## Abstract

Alcohol use disorder (AUD) represents a significant challenge in mental health. Severe AUD is characterized by uncontrolled alcohol consumption and is associated with dysregulation in brain circuits responsible for reward, motivation, decision‐making, affect, and stress response. Mindfulness is known to positively influence those dysregulations and may enhance abstinence‐related self‐efficacy (confidence in resisting alcohol consumption), which is one of the best predictors for abstinence following inpatient treatment. Large‐scale networks underlie mindfulness, including the default mode and salience network. This study aims to investigate the role of the cingulum bundle (CB) in patients with AUD, which bridges these two networks in relation to mindfulness and abstinence‐related self‐efficacy. We conducted a study with 39 recently abstinent inpatients with AUD and 18 healthy controls. Mindfulness and self‐efficacy were assessed using standardized and validated self‐report questionnaires. Structural and resting state functional magnetic resonance imaging (MRI) data were acquired to examine structural and functional connectivity of the cingulate cortex. Our findings showed reduced structural and functional connectivity of the CB in AUD patients with a highly positive association between these metrics. Overall, mindfulness correlated strongly with abstinence‐related self‐efficacy. We found no association of trait mindfulness and structural and functional findings of the cingulate cortex. However, exploratory analyses suggest a positive association between CB number of streamlines and mindfulness factors ‘acceptance’ and ‘decentring’, and abstinence‐related self‐efficacy. This is the first study indicating that patients with AUD have structural and functional impairments of the CB. These alterations could be associated with reduced mindfulness and self‐efficacy.

## Graphical abstract

This study found that patients with alcohol use disorder (AUD) showed reduced structural and functional connectivity in the cingulum bundle (CB), a brain tract linking key networks for self‐regulation. While overall mindfulness was not directly associated with CB connectivity, specific mindfulness components (‘acceptance’ and ‘decentring’) and abstinence‐related self‐efficacy showed positive associations with CB structure. These findings suggest that CB impairments in AUD may contribute to reduced mindfulness and self‐efficacy, which are important for successful abstinence.

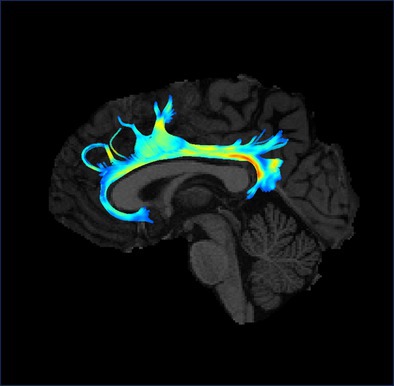

## 1 Introduction

Severe alcohol use disorder (AUD) is a chronic relapsing disorder characterized by difficulties in controlling drinking habits, leading to excessive and compulsive alcohol consumption [1]. The impaired response inhibition and salience attribution (iRISA) model suggests that the inability to inhibit alcohol use is associated with dysregulation of six brain networks, including the reward, habit, salience, executive control, memory, and self‐directed network [2]. Treatment for AUD encompasses various pharmacological and psychological approaches, including motivational enhancement, cognitive behavioural therapy, and other treatments [3, 4, 5].

One promising approach is the implementation of mindfulness‐based interventions to reduce relevant clinical outcome measures such as craving, number of abstinent or heavy drinking days [6]. Mindfulness is a state of consciousness that is characterized by a non‐judgmental way of paying attention to the present moment [7], which positively impacts general mental health and health behaviour [8, 9]. It also improves the differentiation of negative emotions, which may lead to fewer emotional difficulties [10, 11]. Increased focus on mindfulness helps to reduce mind wandering, which refers to a mental state where one's stream of thoughts shifts away from the present task or environment to unrelated internal narratives or daydreams [12]. Mindfulness can be understood as a consistent psychological characteristic, measurable through surveys with proven test–retest reliability [13, 14]. As a trait, it reflects a person's general tendency to maintain awareness and focus on the present moment in various internal and external contexts. Importantly, trait mindfulness can be modified through training and practice, while state mindfulness is a temporary condition (e.g. during meditation). Higher levels of mindfulness are associated with increased scores of emotional awareness [10] but also reduce impulsive behaviour in psychiatric disorders, including AUD [15]. Thus, mindfulness is known to play a significant role in reducing problematic alcohol use and in increasing readiness to change drinking behaviour [16].

Mindfulness has been proven to enhance self‐efficacy [17, 18], which is one of the most important predictors for long‐ and short‐term abstinence and treatment outcome in AUD [19, 20, 21, 22, 23, 24, 25, 26]. Dale et al. [27] examined client characteristics to predict the drinking outcome measured by the percentage of days of abstinence (PDA) and drinks per drinking day (DDD) [27]. Again, the strongest and most consistent predictor of PDA and DDD was self‐efficacy, measured by an adaptation of the Alcohol Abstinence Self‐Efficacy Scale (AASE) [28, 29].

On a neurobiological level, mindfulness is strongly linked to changes in large‐scale networks, including the default mode network (DMN) [30], which refers to the self‐directed network of the iRISA model [2], and the salience network (SN) [31]. A recent meta‐analysis indicates that mindfulness‐based interventions increase functional connectivity (FC) between those two networks through enhanced communication between the posterior cingulate cortex (PCC, part of the DMN) and the anterior cingulate cortex (ACC, part of the SN) [30]. The PCC is involved in self‐referential thoughts, showing reduced activity in mindfulness, which aligns with a decreased focus on the self, and increased activity during mind wandering, reflecting its role in internal and self‐referential processes. The ACC, conversely, plays a crucial role in mindfulness by aiding in self‐regulation, attention, and maintaining focused awareness, but shows decreased activity during mind wandering, indicating reduced attention control [31, 32]. Furthermore, the ACC has structural connections with the striatum, which is a core hub of cortico‐striato‐thalamo‐cortical loops that are essential for motivational processes [33]. These loops are involved in goal‐directed behaviour, reinforcement, and reward processing, and disruptions in this connectivity could lead to difficulties in sustaining motivation or regulating behaviour, particularly in conditions like AUD. The functional consequences of this connectivity highlight how mindfulness may contribute to improved self‐regulation and motivation by modulating these circuits. In our previous analysis, we investigated the role of a thalamocortical system for mindfulness in AUD and found reduced FC between the thalamus and the ACC/PCC. Specifically, reduced thalamus‐PCC FC was associated with diminished mindfulness in patients with AUD [34]. These findings suggest that altered structural and functional connectivity in the cingulate cortex may be linked to deficits in mindfulness and self‐regulation in AUD. In our previous analysis, we investigated the role of a thalamocortical system for mindfulness in AUD and found reduced FC between the thalami and the ACC/PCC. Furthermore, reduced thalamus‐PCC FC was associated with reduced mindfulness in patients with AUD [34].

It is the aim of this study to gain a more comprehensive understanding of the communication within the cingulate cortex, especially structural connectivity (SC) and FC between the ACC and PCC. Furthermore, we are interested in its association with mindfulness and abstinence‐related self‐efficacy in patients with AUD. We assessed SC of the cingulum bundle (CB), a pathway that connects cingular subregions including the ACC and PCC, and ROI‐to‐ROI based FC between the ACC and PCC. We hypothesized reduced SC and FC between ACC and PCC in patients with AUD in contrast to healthy controls. Abstinence‐related self‐efficacy reflects a person's confidence in resisting alcohol use. The CB, connecting the ACC and PCC, supports cognitive control and emotion regulation—key for maintaining sobriety. Stronger structural and functional CB connectivity may enhance self‐monitoring and impulse control, leading to higher self‐efficacy scores. Conversely, reduced CB connectivity may impair these functions, lowering self‐efficacy. Thus, we hypothesized a positive association between CB metrics and trait mindfulness. Furthermore, we calculated exploratory correlations hypothesizing a positive link between mindfulness factors (‘acceptance’, ‘decentring’, ‘openness’), abstinence‐related self‐efficacy and structural and functional cingulum metrics.

## 2 Methods

### 2.1 Participants

Following the baseline assessments that were used for this study, all patients took part in a clinical trial on alcohol‐inhibition training in patients with AUD [35, 36, 37]. This sample overlaps with previous analyses [34, 38, 39, 40] and is identical to the sample used in [38], a subsample with diffusion‐weighted imaging (DWI) scans. The sample includes 39 patients attending a 12‐week abstinence‐oriented residential treatment program for AUD in a specialized treatment centre in Switzerland (Clinic Suedhang). To avoid the confounding factor of atrophy related to age, only participants between 18 and 59 years of age were included. Further inclusion criteria were a primary diagnosis of AUD according to the Diagnostic and Statistical Manual of Mental Disorders, Fifth Edition (DSM‐5; [41]), right‐handedness, and abstinence from alcohol for at least 4 weeks prior to MRI measurement. Exclusions were severe substance use (other than nicotine) and neurocognitive disorders. The study also involved 18 age and sex counterbalanced, right‐handed healthy controls with non‐problematic drinking behaviour (AUDIT < 8; [42]) and low psychopathology scores (Brief Symptom Check List, BSCL, GSI t‐value ≤ 63; [43]). All participants provided written informed consent and received a reimbursement of 50 Swiss Francs for participation. The study was approved by the local ethics committee (KEK‐number: 2016‐00988) and registered at Clinicaltrials.gov (NCT02968537) and the Swiss National Clinical Trials Portal (SNCTP000002043). Detailed socio‐demographical and clinical sample characteristics are shown in Table 1.

**TABLE 1:** Clinical characteristics of groups. See Bracht and collegues [38].

	AUD patients (*n* = 39)	Healthy controls (*n* = 18)	*p*
**Demography**			
Females/males	15/24	6/12	0.709
Age, years	41.92 (8.55)	40.44 (12.12)	0.645
Years of education	14.21 (3.76)	16.33 (3.46)	0.047 *
BDI‐II	1.21 (0.61)	0.18 (0.19)	< 0.001***
BSCL‐GSI	15.70 (9.17)	n/a	n/a
**Alcohol**			
AUDIT	24.44 (7.25)	4.00 (1.88)	< 0.001 ***
Number of detoxifications	2.89 (2.92)	n/a	n/a
Days of abstinence	30.32 (14.65)	n/a	n/a
Years of problematic drinking	11.25 (9.17)	n/a	n/a
**Mindfulness (CHIME)**	3.94 (0.58)	4.35 (0.44)	0.012 *
Inner awareness	4.50 (1.14)	4.89 (0.69)	0.163
Outer awareness	4.71 (1.01)	4.99 (0.90)	0.183
Acting with awareness	4.42 (0.79)	4.31 (0.83)	0.630
Acceptance	3.13 (0.93)	4.00 (0.92)	0.003 **
Decentring	3.62 (0.98)	4.31 (0.63)	0.008 **
Openness	3.17 (0.91)	4.06 (0.82)	< 0.001 ***
Relativity	4.01 (0.82)	40.7 (0.94)	0.795
Insight	3.97 (0.71)	4.19 (0.82)	0.318
**Self‐efficacy (AASE)**			
Relapse temptation	24.51 (18.73)	n/a	n/a
Abstinence confidence	53.77 (18.95)	n/a	n/a
Self‐efficacy	−29.26 (36.32)	n/a	n/a

Abbreviations: AASE, Alcohol Abstinence Self‐efficacy Scale; AUDIT, Alcohol Use Disorders Identification Test; BDI‐II, Beck Depression Inventory; BSCL‐GSI, global severity index of the Brief Symptom Check List; CHIME, Comprehensive Inventory of Mindfulness Experiences; n/a, not applicable.

### 2.2 Questionnaires and Interviews

AUD diagnosis was verified using the Diagnostic Expert System for Psychiatric Disorders (DIA‐X; [44], the AUD part adapted to DSM‐5) and the self‐rating Alcohol Use Disorders Identification Test (AUDIT) to estimate the severity of drinking problems [45]. Alongside alcohol‐specific variables, depressive symptoms were assessed using the Beck Depression Inventory (BDI‐II; [46]), and general clinically significant symptoms were assessed by the global severity index (GSI, mean of all scores) of the Brief Symptom Check List (BSCL; [47]).

### 2.3 Assessment of Mindfulness

The Comprehensive Inventory of Mindfulness Experiences (CHIME) was utilized to measure mindfulness [13, 14]. This inventory comprises 37 items, each rated on a scale from 0 (never true) to 4 (always true). These items are grouped into eight mindfulness factors: 1) ‘inner awareness’ (focusing on internal experiences), 2) ‘outer awareness’ (focusing on external experiences), 3) ‘acting with awareness’, 4) ‘acceptance’ (adopting a non‐judgmental stance), 5) ‘decentring’ (ability to observe without reacting), 6) ‘openness’ (openness to experiences, 7) ‘relativity of thoughts’, 8) ‘insight’ (gaining deeper understanding) and a total score (mean of 8 CHIME factors). The CHIME has demonstrated strong internal consistency, evidenced by a Cronbach's α of 0.84 in the aforementioned studies.

### 2.4 Assessment of Abstinence Self‐Efficacy

For the measurement of self‐efficacy in relation to alcohol abstinence, we used the Alcohol Abstinence Self‐efficacy Scale (AASE), which has shown high reliability and validity with a Cronbach's α of 0.94 [48]. It is a self‐report scale, assessed on a 5‐point Likert scale (0 = not at all to 4 = extremely), of perceived ‘confidence’ and ‘ability’ to abstain from alcohol across 20 different situations [29]. Sum scores are calculated separately for ‘relapse temptation’ and ‘abstinence confidence’. A composite ‘self‐efficacy’ scale is created by subtracting ‘relapse temptation’ from ‘abstinence confidence’. Higher scores indicate higher self‐efficacy to abstain from drinking.

### 2.5 Acquisition of Magnetic Resonance Imaging (MRI)

For the acquisition of MRI data, we used a 3‐T MAGNETOM Prisma scanner (Siemens Healthineers, Erlangen, Germany) with a 64‐channel head and neck coil at the Swiss Institute for Translational and Entrepreneurial Medicine (SITEM) associated with the University Hospital of Bern in Switzerland. Anatomical data were acquired with a high‐resolution 3D T1‐weighted and bias‐field corrected MP2RAGE sequence. Image parameters were voxel size = 1 × 1 × 1 mm^3^, number of slices = 256, matrix = 256 × 256, FOV = 256 × 256 mm^2^, TE = 2.93 ms, TI = 700 and 2500 ms, TR = 5000 ms, resulting in an INV1 and INV2 image. A T1‐weighted UNI volume was generated from the INV1 and INV2 images within the scanner.

We acquired DWIs using a spin‐echo echo‐planar sequence with 64 non‐collinear directions (b‐value = 1000 s/mm^2^) and one b0 image (b0 = 0 s/mm^2^). Parameters were voxel size = 2.2 × 2.2 × 2.2 mm^3^, number of slices = 60, matrix = 128 × 128, FOV = 269 × 269 mm^2^, TR = 6200 ms, TE = 69 ms.

Functional data were acquired using a 6 min 30 s lasting resting‐state fMRI scan performed by whole brain echo planar imaging (EPI). For the acquisition of a resting‐state fMRI with a duration of 6 min 30 s long, we used whole brain echo planar imaging (EPI). Image parameters were volumes = 300, voxel size = 2.2 × 2.2 × 2.2 mm^3^, number of slices = 60, matrix = 104 × 104, FOV = 230 × 230 mm^2^, TE = 37 ms, TR = 1300 ms. Scans were acquired with condition eyes closed and participants were instructed not to ruminate.

### 2.6 Structural Connectivity of the Cingulum Bundle (CB)

**FIGURE 1:**
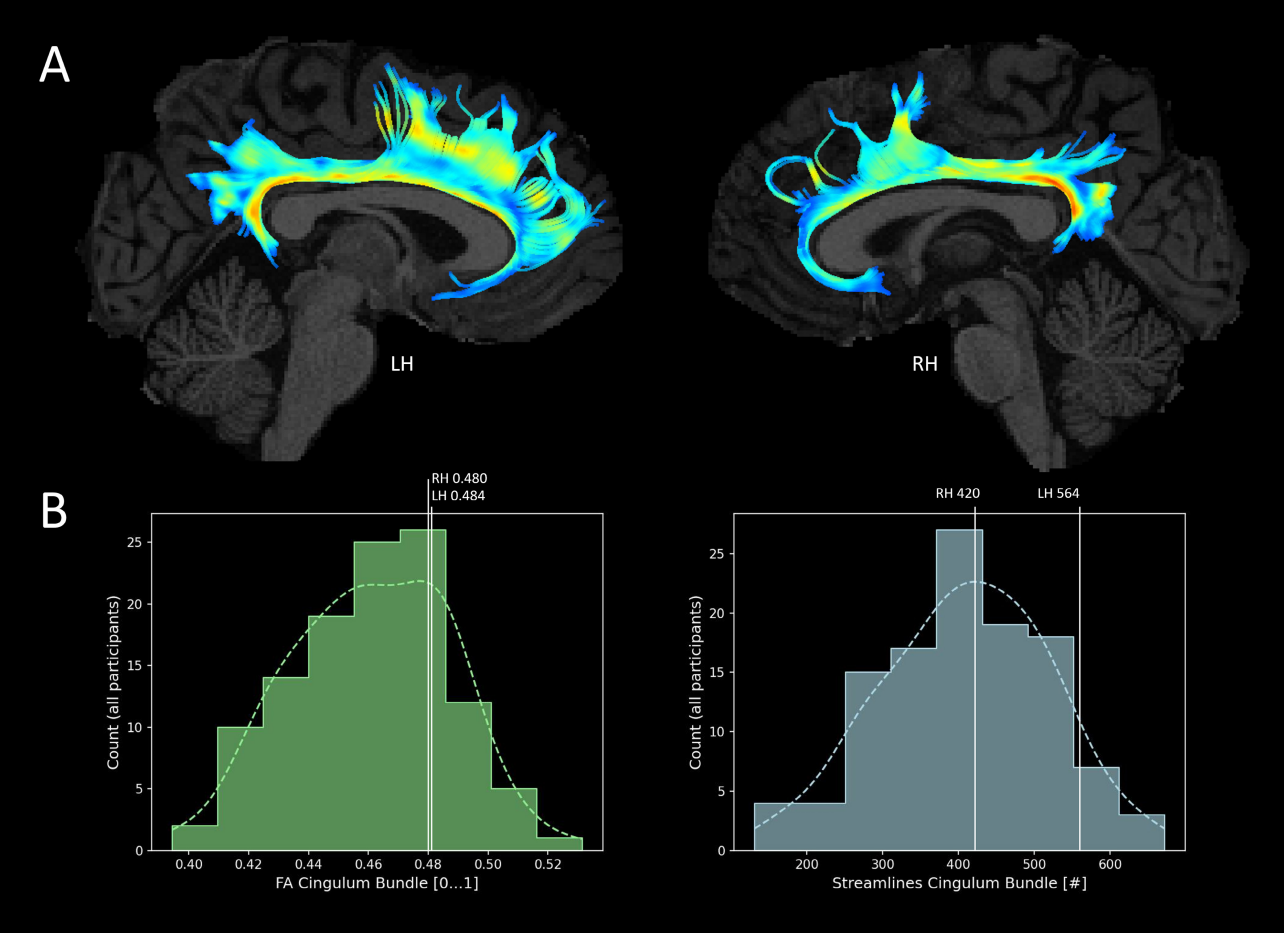
Tractography of the cingulum bundle (CB). (A) Tractography results for bilateral cingulum bundle are displayed for a sample subject in native space. (B) Extracted FA and number of streamlines values of the sample subject are displayed in relation to variability of CB characteristics of all participants. FA, fractional anisotrophy; LH, left hemisphere; RH, right hemisphere.

We used DWI to compute the diffusion tensor, which was used for the tractography of the cingulum bundle. First, we used FSL and FSL‐BET (FSL 6.0, http://www.fmrib.ox.ac.uk/fsl/) to perform robust brain extraction from INV2 images. Binary masks were then applied to UNI images. Second, for the computation of the diffusion tensor out of DWI, we used ExploreDTI 4.8.6 [49]. First, we applied a subject motion and distortion correction to DWI data (reference volume: T2‐weighted b0 image). Second, we warped the resulting data to the brain‐extracted UNI volumes using an EPI correction [50]. We performed whole‐brain deterministic tractography with a diffusion tensor model [51]. Tract termination criteria were set to fractional anisotropy (FA) < 0.2 and tract angle > 45°. Third, for extraction of the bilateral CB, we proceed as described in previous works [52, 53, 54]. To reconstruct left and right CB, we placed one AND gate five slices anterior and one AND gate five slices posterior of the rostro‐caudal midpoint of the body of the corpus callosum. We computed mean bilateral FA [0–1], sampled over left and right CB, as a measure of white matter microstructure and number of streamlines as a marker of fibre geometry. See Figure 1.

### 2.7 Functional Connectivity of the ACC and PCC

We assessed the FC of the ACC‐PCC connection using the *CONN 22a* toolbox [55]. As described in previous publications [34], we first realigned EPI volumes and applied a field map correction. We used the Artefact Detection Tools (ART) toolbox to perform scrubbing of outlier scans (higher than the 98th percentile in framewise displacement [FD] or global BOLD signal). We also computed for each participant the mean FD of motion parameters and mean DVARS, which is the spatial root mean square of the BOLD signal after temporal differencing [56]. We excluded one subject with a mean FD of 0.36 and a mean DVARS of 12.68. The realigned and cleaned EPI volumes were realigned to UNI volumes. Both volumes were then transformed to standard MNI space and smoothed with an FWHM kernel of 8 × 8 × 8 mm. In order to eliminate physiological noise and focus on low‐frequency brain activity, we applied a band‐pass filter with a frequency range of 0.008 to 0.09 Hz to the time series of each voxel in the normalized images. Nuisance variables were regressed using the five timeseries within the segmented white matter and cerebrospinal fluid, as well as the 12 realignment parameters. For every subject, we computed the mean FD of the motion parameters and mean DVARS, which is the spatial root mean square of the BOLD signal after temporal differencing. HC and AUD patients did not differ significantly for mean FD (HC 0.23 ± 0.06, range 0.14–0.33; AUD 0.26 ± 0.08, range 0.13–0.6; *p* = 0.14; 1) and mean DVARS (HC 3.22 ± 0.87, range 1.86–5.15; AUD 3.37 ± 1.93, range 1.64–12.68; *p* = 0.76). To further analyse FC of the ACC and PCC, we computed seed‐based whole‐brain FC maps, with separate ACC and PCC as seed regions, by calculating Pearson correlation coefficients of the BOLD time series. Computed by Pearson correlation coefficients of the BOLD time series.

### 2.8 Statistical Analysis

To analyse group differences, we used the Statistical Package for Social Sciences SPSS 29.0 (SPSS, Inc., Chicago, Illinois). We calculated two separate mixed‐model ANCOVAs for CB metrics (FA, number of streamlines) applying a Bonferroni correction for multiple comparisons (α = 0.05/2 = 0.025, *p*_corr_ = *p* × 2). Group (AUD and HC) was entered as an independent variable, CB metric as the dependent variable and hemisphere (left and right) as the within‐subject factor variable. We controlled for age, sex and years of education. In case of a significant main group effect, post hoc ANCOVAs controlling for age, sex and years of education were calculated. In case of a significant group × hemisphere interaction, we tested each hemisphere separately; otherwise, we used the mean value. All tests were two‐tailed and effect sizes were reported as η^2^ [57].

To analyse the ACC‐PCC connection, we performed a ROI‐to‐ROI FC analysis with ROIs derived from the Automatic Anatomical Labelling (AAL) atlas [58]. We compared HC and AUD using two‐sample *t* test statistics for functional network connectivity [59]. We applied a connection threshold of *p* < 0.05 and a cluster‐level FDR correction of *p* < 0.05. To further analyse the connectivity of the ACC and PCC, we computed seed‐based whole‐brain FC maps, by calculating Pearson correlation coefficients with the mean BOLD time series within the seed. We used separate ACC and PCC (AAL masks) as seed regions. We applied a connection threshold of *p* < 0.01 and a cluster‐level FDR correction of *p* < 0.05.

To investigate possible intermodal association of structure and function of the cingulum, we performed three Spearman r correlations (FA and FC, FA and streamlines, FC and streamlines) within each group (HC and AUD) and applied a Bonferroni correction (α = 0.05/3 = 0.0125, *p*_corr_ = *p* × 3). In order to investigate group differences between the correlations, we proceeded as follows using Python scripting. First, we converted the Spearman *r* correlation coefficients into *z* values using Fisher's *t*‐*z* transformation: $z=0.5\ln\frac{1+r}{1-r}$. Then, we calculated the standard error (SE) of the difference between the two *z* values, where $n_{\mathrm{AUD}}$ and $n_{\mathrm{HC}}$ are the sample sizes: $\mathrm{SE}=\sqrt{1/n_{\mathrm{AUD}}-3/n_{\mathrm{HC}}-3}$. Finally, the difference between z‐scores were divided with the SE ($z_{\text{diff}}=\frac{z_{\mathrm{AUD}}-z_{\mathrm{HC}}}{\mathrm{SE}}$) transformed into *p* values using the cumulative distribution function of NumPy.

To test whether mindfulness is related to structure and function of the CB, we used Spearmen r correlations between mindfulness (CHIME total) and CB FA, CB number of streamlines and CB FC. Due to significant differences in CHIME total correlations were calculated separately for each group (AUD, HC). We performed a Bonferroni correction of α = p/3. In addition, we performed exploratory Spearman r analyses regarding mindfulness factors that were significantly reduced in AUD, abstinence self‐efficacy and CB characteristics (FA, number of streamlines, ROI‐to‐ROI FC) within AUD patients and where appropriate (no significant group differences in MRI metrics) in all subjects.

## 3 Results

### 3.1 Study Population

Sociodemographic and clinical characteristics of patients with AUD and HC are shown in Table 1. The groups did not differ regarding age or sex but did show differences in years of education (HC 16 ± 3 years; AUD 14 ± 4 years; *p* = 0.047) and BDI‐II score (HC 0.2 ± 0.2; AUD 1.2 ± 0.6; *p* < 0.001). AUD patients had significantly lower CHIME total scores than HC (HC 4.4 ± 0.4; AUD 3.9 ± 0.6; *p* = 0.012). CHIME factors were significantly lower in AUD in contrast to HC (*p* < 0.01).

### 3.2 Structural Connectivity of the Cingulum Bundle (CB)

**FIGURE 2:**
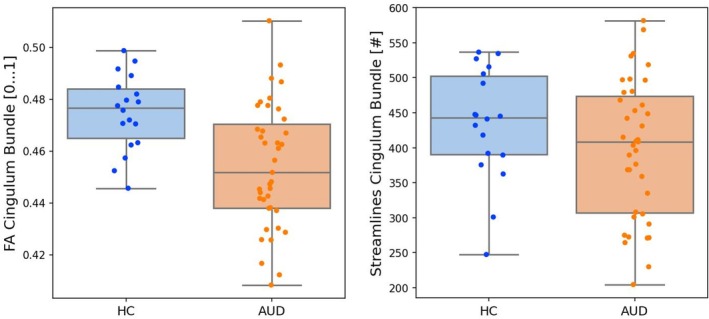
Altered DTI metrics of the cingulum bundle (CB) in patients with AUD compared to healthy controls (HCs). Boxplots of group differences of FA and number of streamlines of the CB.

Comparing AUD patients with HC regarding FA values of the CB, the mixed model ANCOVA revealed a significant main group effect (*F*_(1,52)_ = 11.415, *p* = 0.0014, *p*_corr_ = 0.0028, large effect size η^2^ = 0.180). There was no significant group × hemisphere interaction (*F*_(1,52)_ = 2.391, *p* = 0.128, η^2^ = 0.04). Post hoc tests revealed significantly reduced FA in AUD as compared to HC in the bilateral CB (AUD 0.454 ± 0.023 vs. HC 0.475 ± 0.015, *F*_(1,52)_ = 11.415, *p* = 0.0014, large effect size η^2^ = 0.180). Comparing AUD patients with HC regarding number of streamlines of the CB, the mixed model ANCOVA revealed no significant main group effect (*F*_(1,52)_ = 1.351, *p* = 0.250, η^2^ = 0.025) and no significant group × hemisphere interaction (*F*_(1,52)_ = 1.499, *p* = 0.226, η^2^ = 0.028). See Figure 2.

### 3.3 Functional Connectivity of the ACC and PCC

**FIGURE 3:**
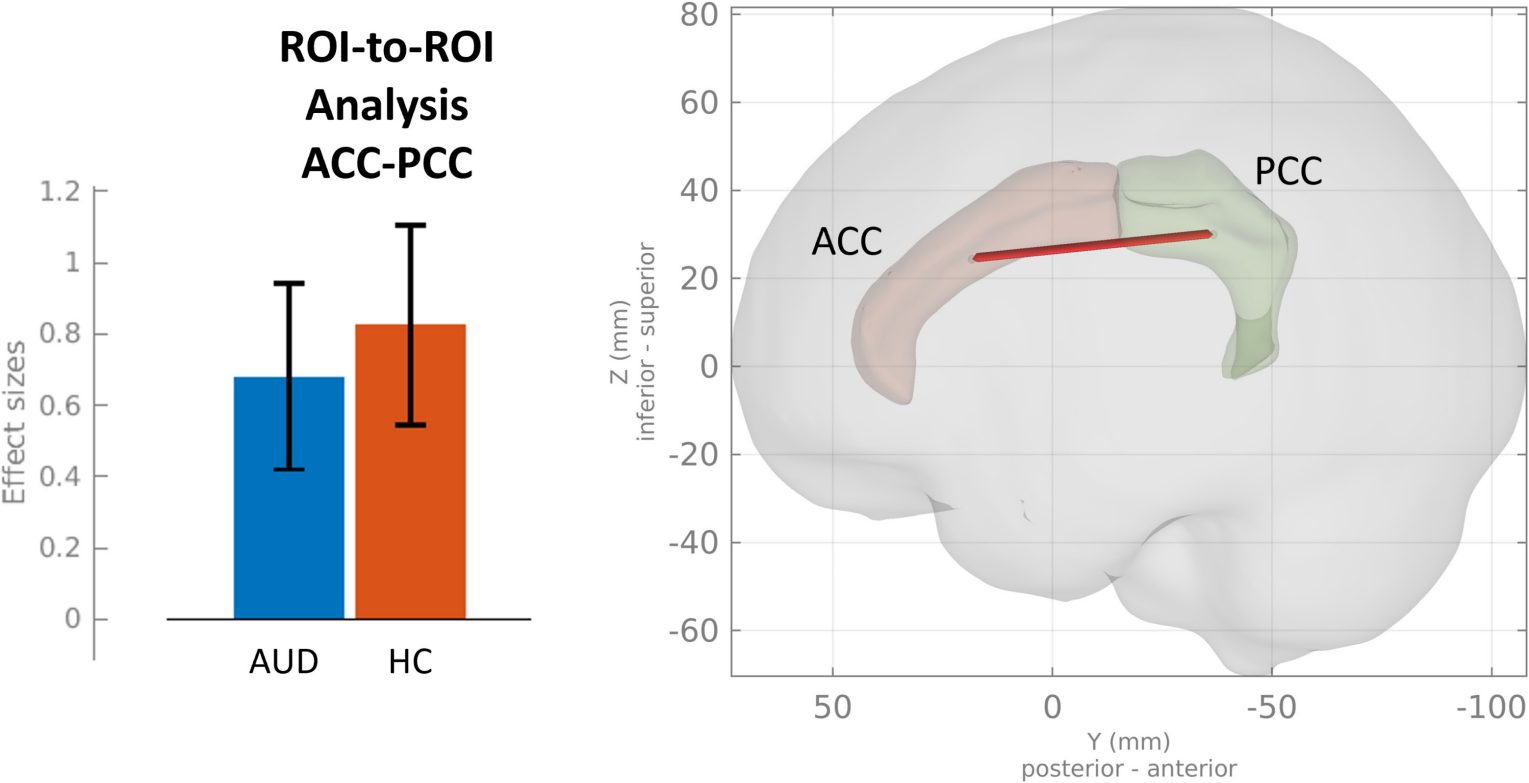
Lower ROI‐to‐ROI based functional connectivity (FC) between the anterior and posterior cingulate cortex in patient with AUD compared to healthy controls (HCs). ACC, anterior cingulate cortex; PCC, posterior cingulate cortex.

Patients with AUD showed reduced ROI‐to‐ROI FC between the ACC and PCC compared to HC (*F*_(1,52)_ = 6.01, p‐FDR = 0.018). See Figure 3. We found no significant clusters in group comparisons regarding seed‐based FC of the ACC and PCC.

### 3.4 Associations Between Structural and Functional MRI Metrics

Within patients with AUD, FA of the CB and ROI‐to‐ROI FC correlated significantly (*r* = 0.457, *p* = 0.003, *p*_corr_ = 0.009). CB's FA and numbers of streamlines correlated significantly (*r* = 0.421, *p* = 0.008, *p*_corr_ = 0.024). Within HC, there were no significant correlations between cingulum metrics. There were no significant group differences in intermodal correlations. See Table 2.

**TABLE 2:** Intermodal correlations of cingulum metrics.

	FA of CB	Streamlines in CB	FC of ACC‐PCC
**FA of CB**	1		
**Streamlines in CB**	AUD: *r* = 0.42, *p* = 0.008* HC: *r* = 0.44, *p* = 0.07 Diff: *z* = −0.07, *p* = 0.47	1	
**FC of ACC‐PCC**	AUD: *r* = 0.46, *p* = 0.003* HC: *r* = 0.04, *p* = 0.87 Diff: *z* = 1.47, *p* = 0.93	AUD: *r* = 0.11, *p* = 0.51 HC: *r* = −0.10, *p* = 0.70 Diff: *z* = 0.67, *p* = 0.75	1

Abbreviations: ACC, anterior cingulate cortex; Diff, normalized group differences in *z* values; FA, fractional anisotropy; FC, functional connectivity; PCC, posterior cingulate cortex; *r*, correlation coefficient.

### 3.5 Associations of Cingulum Metrics, Mindfulness and Abstinence Related Self‐Efficacy

We found no significant correlation of mindfulness (CHIME total) and cingulum metrics in HC and AUD. See Table 3.

**TABLE 3:** Association of mindfulness and cingulum metrics.

Correlation with mindfulness (CHIME total)	AUD patients (*n* = 39)	Healthy controls (*n* = 18)
FA cingulum bundle	*r* = 0.096, *p* = 0.563	*r* = −0.282, *p* = 0.257
Streamlines cingulum bundle	*r* = 0.690, *p* = 0.677	*r* = 0.197, *p* = 0.433
FC of ACC‐PCC	*r* = 0.105, *p* = 0.533	*r* = −0.032, *p* = 0.900

Abbreviations: ACC, anterior cingulate cortex; CHIME, Comprehensive Inventory of Mindfulness Experiences; FA, Fractional anisotropy; FC, functional connectivity; PCC, posterior cingulate cortex.

**FIGURE 4:**
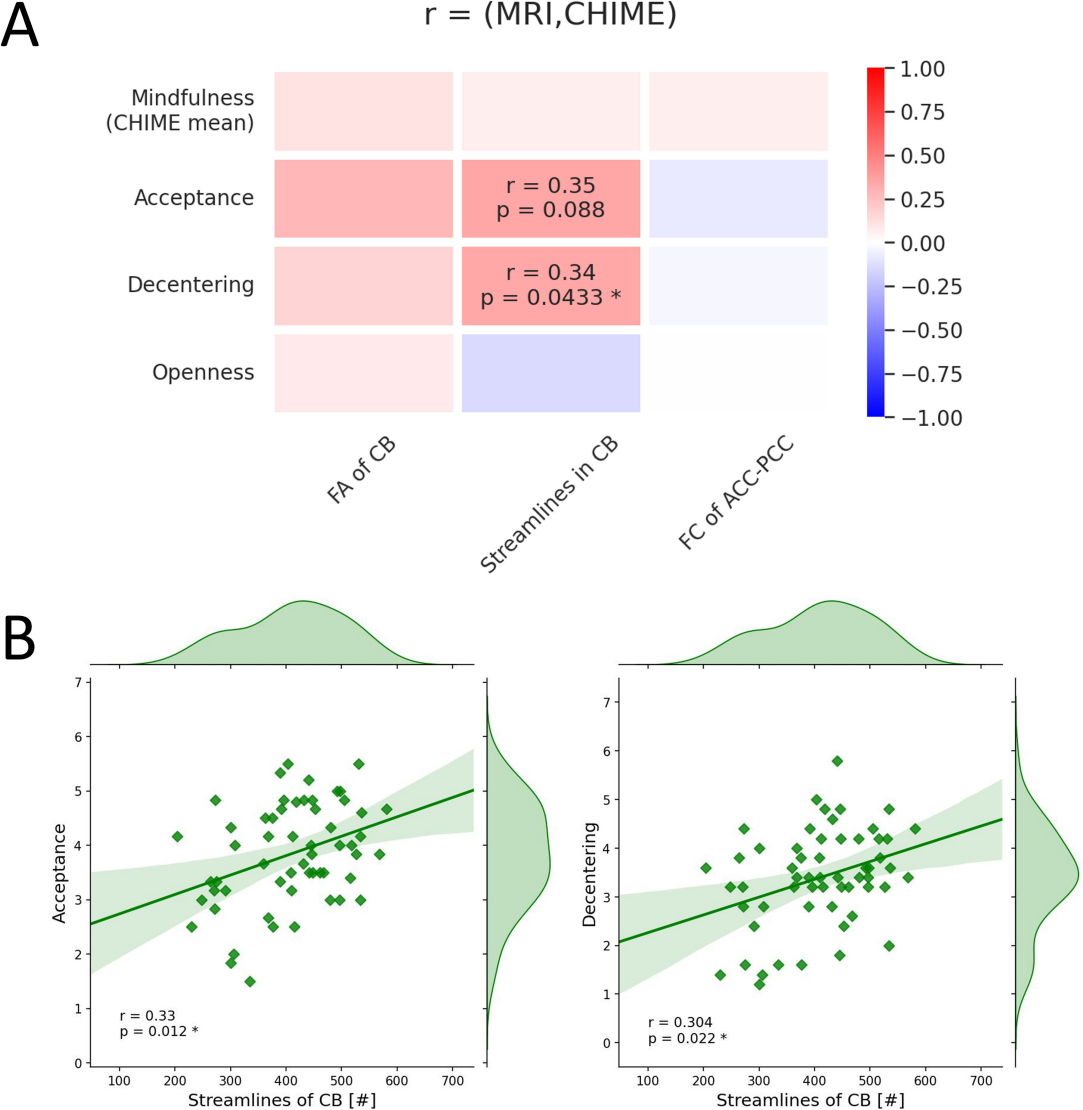
Exploratory correlations of significant mindfulness factors (CHIME) and extracted MRI data. (A) Exploratory correlations within AUD patients. Only values with *p* < 0.1 are shown numerically. (B) Exploratory correlations of CB streamlines (no significant group difference) and mindfulness factors ‘acceptance’ and ‘decentring’ within all participants. FA, fractional anisotropy; FC, functional connectivity.

**FIGURE 5:**
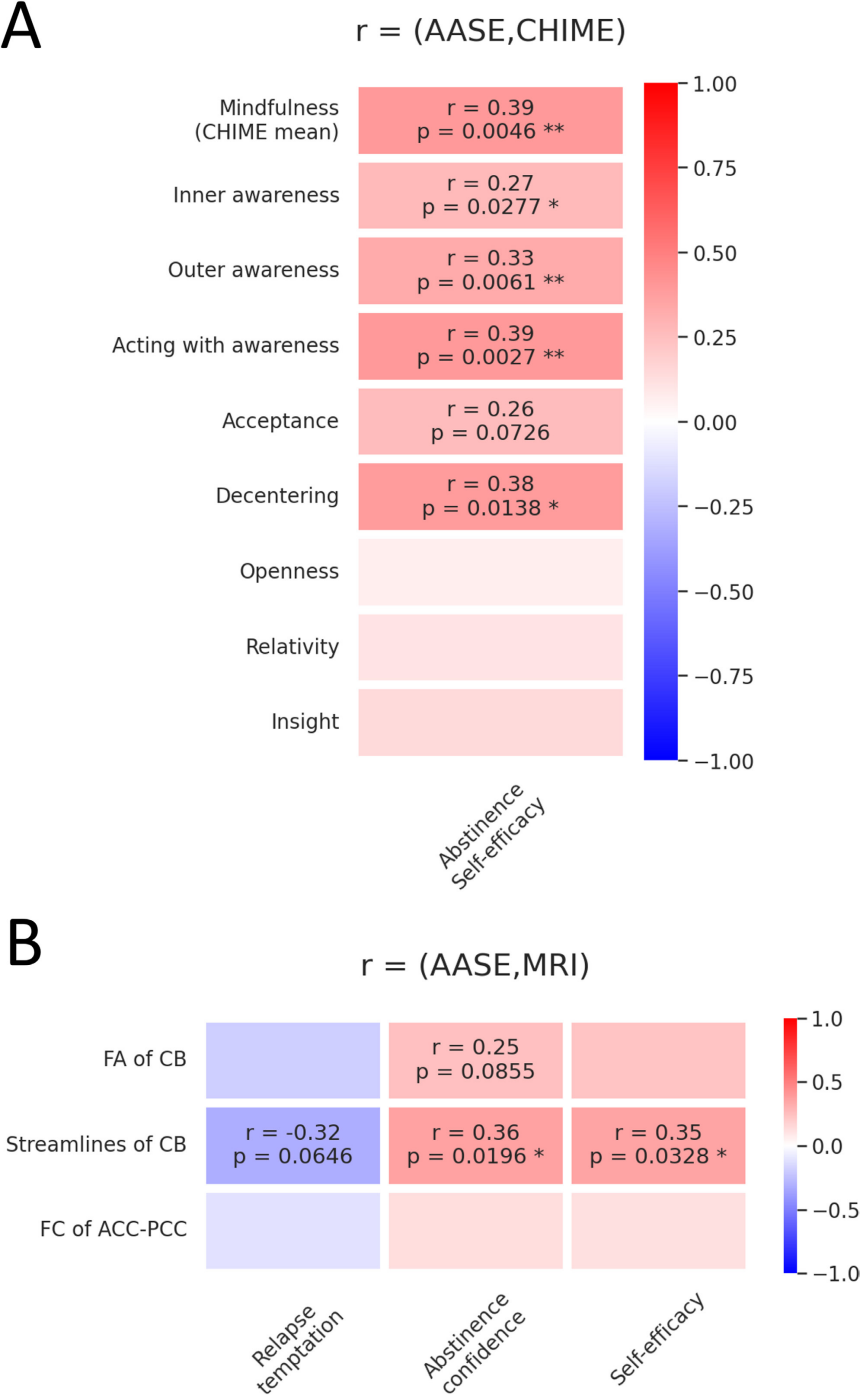
Exploratory correlations of the Alcohol Abstinence Self‐efficacy Scale (AASE) in relation to mindfulness and cingulum bundle (CB) metrics within patients with AUD. (A) Exploratory correlations of AASE and mindfulness factors. (B) Exploratory correlations of AASE and extracted MRI values. Only values with *p* < 0.1 are shown numerically. FA, fractional anisotropy; FC, functional connectivity.

Exploratory correlations of extracted MRI data revealed a positive association between number of streamlines of the left CB and mindfulness factors ‘decentring’ (*r* = 0.34, *p* = 0.043) and ‘acceptance’ (*r* = 0.35, *p* = 0.088, non‐significant trend) within AUD patients and a positive association of number of streamlines of the left CB and mindfulness factors ‘acceptance’ (*r* = 0.33, *p* = 0.012) and ‘decentring’ (*r* = 0.304, *p* = 0.022) within all subjects. None of these significant correlations would survive a multiple comparison correction. See Figure 4A,B. Exploratory correlations of mindfulness (overall and factors ‘inner awareness’, ‘outer awareness’, ‘acting with awareness’, ‘acceptance’, and ‘decentring’) revealed significant positive associations with AASE self‐efficacy in patients with AUD. By using a Bonferroni correction of *p*_corr_ = 0.05/9 = 0.0056, only overall mindfulness (*r* = 0.39, *p* = 0.0046) and ‘acting with awareness’ (*r* = 0.39, *p* = 0.0027) would remain significant. See Figure 5A. Furthermore, number of streamlines of the CB correlated significantly with AASE ‘self‐efficacy’ (*r* = 0.35, *p* = 0.033) and ‘abstinence confidence’ (*r* = 0.36, *p* = 0.02) in the patient group. There was a negative trend association of number of streamlines and ‘relapse temptation’, and a positive trend of CB FA and ‘abstinence confidence in patients with AUD’. None of these correlations would survive a multiple comparison correction. See Figure 5B.

## 4 Discussion

This is the first study that investigated structural and functional characteristics of the cingulate cortex in patients with AUD. We found reduced SC, as measured by FA in bilateral CB and reduced ROI‐to‐ROI based FC of the ACC/PCC in patients with AUD, compared with a healthy population. These intermodal metrics were highly positively correlated among patients. In contrast to our a priori hypothesis, we found no positive association of mindfulness total score and cingulum metrics. However, exploratory analyses revealed that fibre geometry (number of streamlines) correlated with mindfulness factors (‘decentring’ and ‘acceptance’) and with abstinence‐related self‐efficacy.

Self‐efficacy correlated also positively with various mindfulness factors, including those that showed a significant group difference when compared to healthy controls (overall mindfulness, ‘decentring’, and a positive trend for ‘acceptance’).

The CB is a major connection pathway between the ACC, a core hub of the SN [31], and the PCC, a central region of the DMN [30]. Both SN and the DMN are important to achieve and maintain states of mindfulness. Also, both networks are altered in AUD [2] and activation in their central nodes—ACC and PCC—has been related to cue‐reactivity, craving and difficulties in alcohol‐specific inhibition [60, 61, 62, 63]. Therefore, one may assume that structural impairments in the CB affect a functioning interaction between the ACC and the PCC, hereby leading to functional impairments, which may also manifest itself in reduced mindful behaviour. Our finding of reduced FA of the CB in AUD complements previous studies investigating patients with AUD [64], including tractography studies of the CB [65] and studies that linked SC deficits of the CB to executive functioning [66]. Our study extends these findings by using a multimodal imaging approach. In addition to alterations of SC in the CB, we found reduced FC between the ACC and the PCC. This also supports the assumption of a disturbed interplay between the DMN and SN. Altered communication within the DMN and SN has already been observed in patients with AUD [67, 68, 69]. Further investigations of between network communication and dynamics (entropy and complexity) of modular networks may help to understand information processing in the brain [70].

One may speculate that toxic effect of alcohol disrupts structural connectivity within the brain, including the CB, which may result in behavioural changes. Indeed, our findings suggest that reduced streamlines of the left CB are associated with lower scores of the mindfulness factors ‘acceptance’ and ‘decentring’. These two factors, together with ‘acting with awareness’, were significantly reduced compared to healthy controls and associated with abstinence self‐efficacy. Noteworthy, reductions of FA of the CB and FC were associated with more pronounced reductions of FC between the ACC and PCC. Thus, one may assume that impairments of SC of the CB disrupt the interplay of the ACC and the PCC, core regions for mediating mindfulness.

Self‐efficacy is a clinically relevant behaviour‐specific construct that plays a major role in the treatment of various disorders, including AUD. The perception of one's ability to moderate or abstain from alcohol use predicts outcomes following AUD treatment. There is evidence that high self‐efficacy is one of the most important predictors of abstinence following treatment in patients with AUD [19, 21, 22, 23, 25, 26]. A recent study examining the connection between self‐efficacy and mindfulness in a non‐clinical sample showed that self‐efficacy worked as a partial mediator in the association between mindfulness, stress, depression and anxiety symptoms [71]. This is in line with a recent study investigating the effect of mindfulness on stress and self‐efficacy in substance use disorder, showing that patients with high levels of mindfulness were found to have low levels of stress and high levels of self‐efficacy [72]. This is backed up by a study in patients with substance use disorders, showing that patients attending a mindfulness‐based relapse prevention program showed the highest self‐efficacy and fewer heavy drinking days at 12‐month follow‐up compared to patients receiving cognitive behavioural therapy or treatment as usual [73]. Decentring is a key component of mindfulness that involves being able to separate from emotions and adopt a non‐judgmental view. Studies have shown that as the ability to decentre increases, anxiety levels decrease [74]. Decentring is closely related to acceptance and non‐attachment, and these three constructs are considered core themes in mindfulness‐based interventions [75]. Decentring plays a role in the efficacy of mindfulness‐based interventions for reducing symptoms of depression and preventing relapse [76]. Overall, acceptance and decentring are important components of mindfulness that have been associated with various positive outcomes, including reduced anxiety, depression symptoms and preventing relapse.

High rates of relapse highlight the need for long‐term treatment provided through different modalities and settings according to the severity of AUD. The relapsing nature of severe AUD has made it clear that relapse prevention is an essential treatment component for long‐term recovery, shifting attention to interventions that address this need, such as mindfulness‐based treatment approaches [77]. In the context of addiction treatment, mindfulness can promote awareness of external and internal triggers for addictive behaviours and improve tolerance to uncomfortable emotional, cognitive and physical experiences [78]. It can also have a significant effect on decreasing craving after treatment [79]. Mindfulness‐based relapse prevention (MBRP) was developed by Marlatt [80] and is an intervention that integrates mindfulness meditation with traditional relapse prevention techniques. A recent review by Ramadas and colleagues evaluating the effectiveness of MBRP on patients with substance use disorders indicates that it has a significant positive impact on various substance use and clinical variables [77]. However, the neurobiological effects of MBRP have not yet been investigated. Since MBRP primarily aims to enhance present‐moment awareness, individuals practicing MBRP may likely exhibit changes in the prefrontal cortex and ACC circuitry that align with their level of present‐moment awareness [81].

Finally, the study has some limitations. First, the sample size is relatively small, which may affect the generalizability of the results. However, we observed large effects sizes regarding group comparisons, which may mitigate concerns about statistical power. Second, findings of DTI‐based alterations of SC of the CB does not allow for sub‐compartment specific conclusions regarding neurobiology. Third, the cross‐sectional design does not allow for conclusions on causality regarding the associations between impaired mindfulness, AUD, and alterations of brain structure and function. Longitudinal studies are needed to better understand the directionality and potential causal mechanisms underlying these associations. Additionally, other potential confounding variables, such as medication use or comorbid psychiatric conditions, were not fully controlled for, which could influence the observed results. Fourth, an additional limitation of this study is that exploratory correlations indicated a potential link between streamlines (as assessed through structural connectivity) and mindfulness, even though there were no significant group differences of streamlines between patients with AUD and HC. Furthermore, there was no association between streamlines and FC. This suggests that mindfulness may be associated with brain network microstructure. However, the relevance of this finding for functional connectivity and overall microstructural alterations in patients with AUD may not be as straightforward as anticipated. Fifth, a low resting‐state MRI acquisition duration of six and a half minutes can reduce the stability, reliability and sensitivity of FC measurements, potentially missing subtle interactions between the ACC and PCC.

In sum, our results point to structural and functional impairments of the CB in patients with AUD. More pronounced alterations of fibre geometry of the CB were associated with more pronounced impairments of mindfulness and lower abstinence self‐efficacy in patients with AUD. This suggests that DMN/SN alterations commonly observed in patients with AUD may be associated with reduced mindfulness and self‐efficacy, which in turn is the strongest predictor for treatment outcome in patients with AUD. Future studies are needed to understand MBRP's effects on self‐efficacy and impaired SC and FC of the CB.

## Author Contributions

N.D., L.M.S., and T.B. conceived the idea and methodology of the study and wrote the original draft. N.D. analyzed the MRI data and conducted the statistical analyses. L.M.S., M.S., and M.G. recruited subjects and were involved in clinical and diagnostic assessments and managed the MRI and clinical data. A.F. and R.W. provided technical support for MRI scanning and data processing. All authors contributed to the article and approved the submitted version.

## Conflicts of Interest

The authors declare no conflicts of interest.

## Data Availability

The data that support the findings of this study are available from the corresponding author upon reasonable request.
